# EXclusion of non-Involved uterus from the Target Volume (EXIT-trial): an individualized treatment for locally advanced cervical cancer using modern radiotherapy and imaging techniques

**DOI:** 10.1186/s12885-018-4800-0

**Published:** 2018-09-17

**Authors:** Katrien Vandecasteele, Philippe Tummers, Mieke Van Bockstal, Pieter De Visschere, Tom Vercauteren, Werner De Gersem, Hannelore Denys, Eline Naert, Amin Makar, Wilfried De Neve

**Affiliations:** 1 Radiation Oncology, Ghent University Hospital, C. Heymanslaan 10, 9000 Ghent, Belgium; 2 Gynaecologic Oncology, Ghent University Hospital, C. Heymanslaan 10, 9000 Ghent, Belgium; 3 Pathology, Ghent University Hospital, C. Heymanslaan 10, 9000 Ghent, Belgium; 4 Pathology, Erasmus Medical Center, s-Gravendijkwal 230, 3015 Rotterdam, The Netherlands; 5 Radiology, Ghent University Hospital, C. Heymanslaan 10, 9000 Ghent, Belgium; 6 Medical Oncology, Ghent University Hospital, C. Heymanslaan 10, 9000 Ghent, Belgium

**Keywords:** Locally advanced cervical cancer, Target volume, Uterus, Diffusion weighted MRI, Apparent diffusion coefficient

## Abstract

**Background:**

Definitive chemoradiotherapy is standard of care in locally advanced cervical cancer (LACC). Both toxicity and local relapse remain major concerns in this treatment. We hypothesize that a magnetic resonance imaging (MRI) based redefining of the radiotherapeutic target volume will lead to a reduction of acute and late toxicity. In our center, chemoradiotherapy followed by hysterectomy was implemented successfully in the past. This enables us to assess the safety of reducing the target volume but also to explore the biological effects of chemoradiation on the resected hysterectomy specimen.

**Methods:**

The EXIT-trial is a phase II, single arm study aimed at LACC patients. This study evaluates whether a MRI-based exclusion of the non-tumor-bearing parts of the uterus out of the target volume results in absence of tumor in the non-high doses irradiated part of the uterus in the hysterectomy specimen. Secondary endpoints include a dosimetric comparison of dose on normal tissue when comparing study treatment plans compared to treatment of the whole uterus at high doses; acute and chronic toxicity, overall survival, local relapse- and progression-free survival. In the translational part of the study, we will evaluate the hypothesis that the baseline apparent diffusion coefficient (ADC) values of diffusion weighted MRI and its evolution 2 weeks after start of CRT, for the whole tumor as well as for intra-tumoral regions, is prognostic for residual tumor on the hysterectomy specimen.

**Discussion:**

Although MRI is already used to guide target delineation in brachytherapy, the EXIT-trial is the first to use this information to guide target delineation in external beam radiotherapy. Early therapy resistance prediction using DW-MRI opens a window for early treatment adaptation or further dose-escalation on tumors/intratumoral regions at risk for treatment failure.

**Trial registration:**

Belgian Registration: B670201526181 (prospectively registered, 26/11/2015); ClinicalTrials.gov Identifier: NCT03542942 (retrospectively registered, 17/5/2018).

## Background

Definitive chemoradiotherapy (CRT) is considered standard of care in Locally Advanced (FIGO 1B2-IVA) Cervical Cancer (LACC). Definitive CRT is a 2-steps process consisting of external beam radiotherapy (EBRT) ± chemotherapy (If possible cisplatin) and a brachytherapeutic (BT) boost. Even with the use of image-guided dose-intensified BT, local relapse arising from CRT-resistant foci is high (3y-local pelvic control rates of 73% up to 96%, depending on stage and treating center) and remains a major cause of treatment failure [1–3]. In exchange for an improved overall survival (OS), adding chemotherapy to conventional EBRT has doubled the risk of severe acute hematological and gastro-intestinal toxicity and tripled platelet toxicity [4].

Triggered off by both the high local recurrence and the toxicity rates we challenged the gold standard by introducing intensity-modulated arc therapy (IMAT) followed by C1 radical hysterectomy (HRT) [5]. This HRT allows removing CRT-resistant tumor foci [6, 7]. IMAT reduces the dose to the surrounding normal organs (organs at risk = OARs) while enabling the delivery of a simultaneously integrated boost (SIB) to the primary tumor and/or enlarged lymph nodes [8]. Such a SIB, which replaces the BT boost, maximally exploits the positive relationship between higher doses, shorter overall treatment time and tumor control [9]. The combination of IMAT and chemotherapy (if possible, cisplatin is called IMAT-C. We reported previously that using IMAT-C safely allows for a post-IMAT-C HRT. Local control and survival rates were promising and both IMAT-C and surgical related toxicity rates were low [6, 7]. Further research aiming at decreasing toxicity and increasing disease control is needed.

We hypothesize that redefinition of the target volume (TV) will lead to a further reduction of acute and late toxicity. Apart from treating the tumor (“gross tumor volume” or GTV) to the highest dose, international guidelines advise to treat organs having a risk of microscopic disease (“clinical target volume” or CTV) also to the same (or high) dose. In case of LACC, the uterus is one of these organs. Influenced by bladder and/or rectal filling and tumor shrinkage, the uterus can tilt from anteflexed to retroflexed position. Angle rotations of 30° and more are noted in up to 1/5 patients [10]. This movement of the uterus and consequently the CTV remains a major problem. Concerns about these CTV movements (with the uterine fundus being the main contributor) have lead to the use of generous margins (up to 4 cm around the uterine fundus) to create the planning target volume or PTV [11]. In this way adequate coverage of the CTV at all time is ensured. Consequently, the volume of irradiated normal tissue is large and the risk of toxicity increases. However, there is no unequivocal evidence supporting the dogma that the whole uterus should be included in the CTV [12]. This “inclusion-dogma” goes back to the pre- magnetic resonance imaging (MRI) era where one could not differentiate the tumor from the uterus. Nowadays, MRI allows discrimination of the tumor with a high level of accuracy [13, 14]. Therefore, we hypothesize that excluding the non-tumor-bearing parts from the CTV, based on the MRI, leads to significant reductions in normal tissue irradiated (OARs) with tighter PTV margins [15] and reduced toxicity. Above that, HRT post-CRT gives us the unique opportunity to explore the biological effects of IMAT-C at the resected HRT specimen.

## Methods/design

### Study design

The EXIT-trial is a phase II, single arm study. The study was initiated by the Department of Radiation Oncology in collaboration with the Departments of Radiology, Pathology, Medical Oncology and Gynaecologic Oncology (Ghent University Hospital, Ghent Belgium).

### Study objectives and endpoints

The objective of this study is to assess the safety of excluding the non-involved uterus from the TV in the definitive CRT treatment of LACC. The primary endpoint is absence of tumor in the non-involved (as determined on the pre-treatment MRI) and non-high doses irradiated part of the uterus in the hysterectomy specimen after CRT. Secondary endpoints include a dosimetric comparison of dose on the OARs when comparing study treatment plans to treatment of the whole uterus at high doses; acute and chronic toxicity, OS, local relapse free and progression free survival (PFS). A translational part of the study is discussed below.

### Inclusion/exclusion criteria

Eligible patients suffer from biopsy proven carcinoma of the uterine cervix. Following inclusion criteria should be met: locally advanced disease (FIGO IB2 or > FIGO IIB or node positive) proven by clinical examination, 18-fluorodeoxyglucose positron emission tomography scan (^18^FDG PET-CT) and MRI; no more than 2 distant metastases (other than para-aortic or inguinal lymph nodes); WHO 0–2; adequate kidney function for cisplatin, in case of inadequate kidney function cisplatin can be replaced by 5-FU or radiotherapy can be the sole therapeutic regimen; not pregnant or breastfeeding; absence of any psychological, familial, sociological or geographical condition potentially hampering compliance with the study protocol and follow-up schedule; willing and able to sign a written informed consent. Patients unable to undergo MRI for any reason are excluded.

### Interventions

**Fig. 1:**
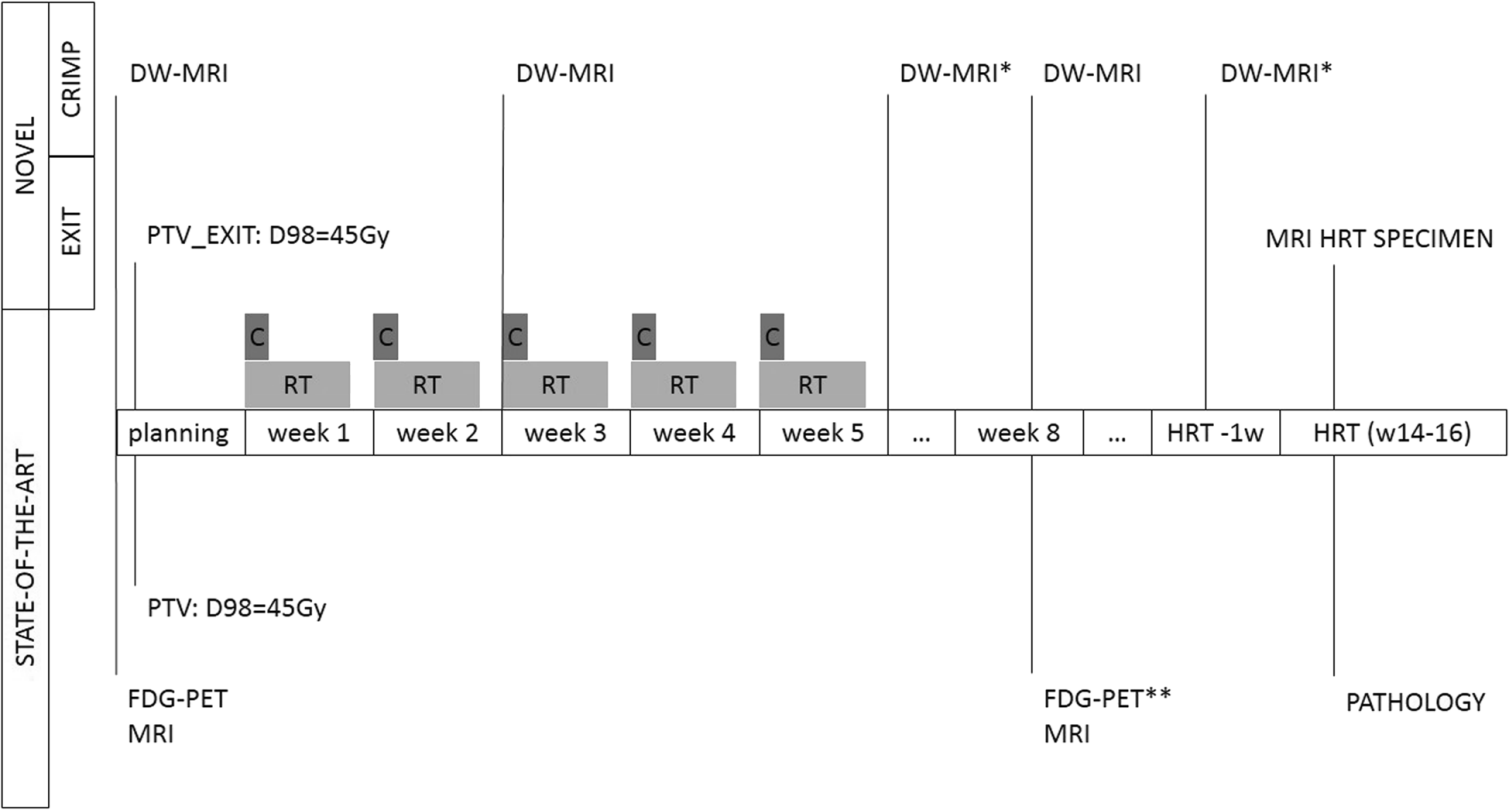
Workflow per patient and situation of the different time-points for each study. DW-MRI: diffusion-weighted MRI; PTV_EXIT: planning target volume as defined within the EXIT-trial; MRI HRT: Magnetic resonance imaging of the HRT specimen; FDG-PET: 18-fluorodeoxyglucose PET scan; PTV: planning target volume; MRI: magnetic resonance imaging. C: weekly chemotherapy; RT: radiotherapy; D98: dose received in 98% of the volume. *: Imaging which requires extra effort of the patient; **: only if node-positive at diagnosis

Figure 1 illustrates the patient-flowchart and all trial-related procedures.

#### Radiation treatment planning

**Fig. 2:**
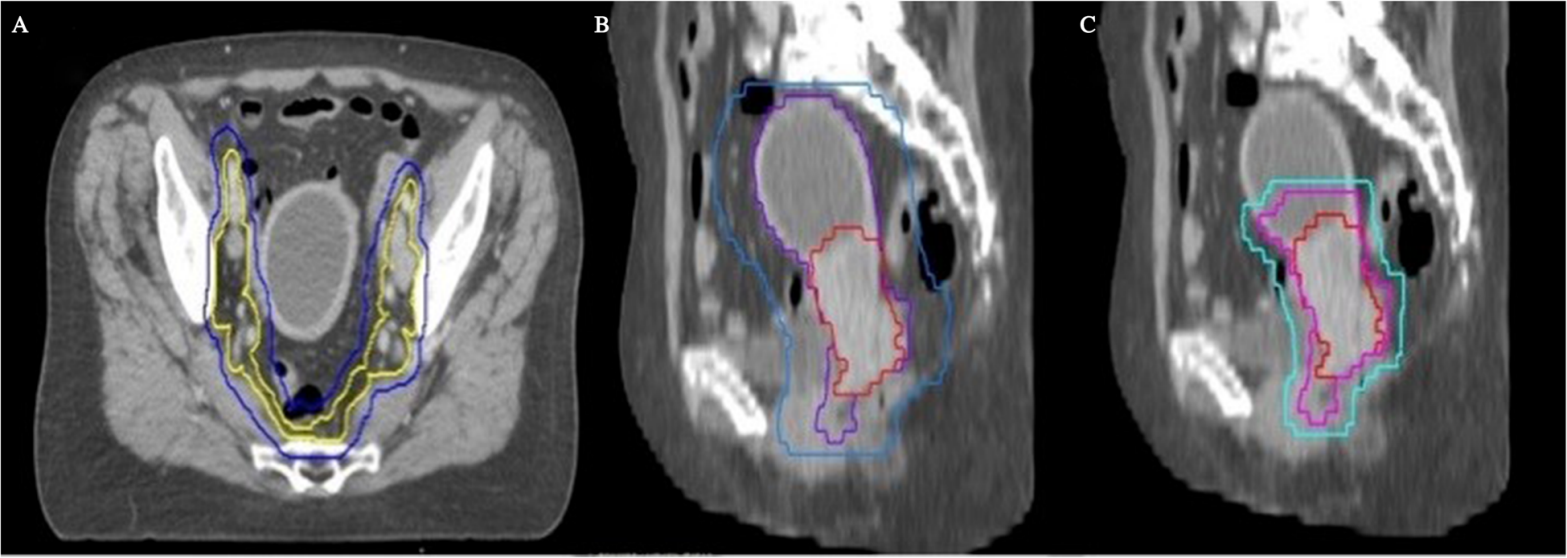
delineation of treatment volumes following state-of-the-art (**a** en **b**) en EXIT-trial (**a** en **c**). **a** delineation of elective lymph node areas are state-of-the-art and identical in standard treatment & the EXIT_trial. Clinical target volume (CTV) of the lymph nodes (lnn) = CTV_Lnn (yellow) = elective lnn areas (common, external and internal lnn, obturator and presacral region; inclusion of para-aortic lnn if any pelvic lnn are affected). Planning target volume (PTV) of the lnn (PTV_Lnn; blue) is created using an isotropic margin of 5 mm around the CTV_Lnn. **b** GTV of the primary tumor (GTV_prim = red): primary tumor delineated using T2 weighted MRI images (state of the art). CTV of the primary tumor (CTV_prim = blue): includes GTV_prim, whole uterus, non-affected parts of the cervix and parametria, upper vaginal 1/3 to ½ (minimal vaginal margin of 2 cm to the GTV_prim). PTV of the primary tumor (PTV_prim = purple): margin around the CTV_prim of 10 mm antero-posterior (AP); 5 mm left-right (LR) and 5 mm supero-inferior (SI)

After imaging in treatment position (standard procedure or state of the art: ^18^FDG PET-CT and MRI as described in Fig. 1) [7, 8] and fusion with the RT-planning-CT, TVs and OARs will be delineated. Planning CT (p-CT) was taken on a Toshiba 16 slice CT scanner, in supine position, using intravenous and bowel contrast. Patients were scanned with a comfortable filled bladder. Delineated OARs include small intestine, rectum, sigmoid colon, bladder and bone marrow. Per patient 2 sets of PTV (see Fig. 2) will be generated, in which the PTV of the lymph nodes (lnn), (=PTV_lnn) is identical:

## PTV: PTV_prim + PTV_lnn (state-of-the-art, as performed out of this trial)

**PTV_prim** is created by an anisotropic expansion around the CTV of the primary (prim) tumor (= CTV_prim) of 10 mm antero-posterior (AP); 5 mm left-right (LR) and 5 mm supero-inferior (SI). CTV_prim consists of the primary tumor delineated using T2 weighted MRI images (GTV_prim) + whole uterus + non-affected parts of the cervix and parametria + upper vaginal 1/3 to ½ (minimal vaginal margin of 2 cm to the GTV_prim).

**PTV_lnn** is created using an isotropic margin of 5 mm around the CTV_lnn (CTV of the lnn) which consists of the union of following regions: common-, external- and internal iliac lnn, obturator and presacral region and para-aortic lnn if any pelvic lnn are affected. A lymph node is considered affected when.

## PTV_EXIT: PTV_prim_EXIT + PTV_Lnn

**PTV_prim_EXIT** is created using an anisotropic margin around the CTV_prim_EXIT of 7 mm AP; 5 mm LR and 5 mm SI. CTV_prim_EXIT consists of the GTV_prim + uterus within 1,2 cm around GTV_prim [16] + non-affected parts of the cervix and parametria + upper vaginal 1/3 to ½ (minimal margin of 2 cm around the GTV_prim). The **PTV_lnn** is identical as discussed above.

A treatment plan is made for both TVs using the Raysearch planning system (RaySearch Laboratories, Stockholm, Sweden) using identical prescription and constraints as in our previously published work [7, 8]. In short, a minimal dose (D98) of 45 Gy in 25 fractions is prescribed to the PTV and PTV_EXIT. A minimal dose (D98) of 62Gy is prescribed to the GTV_prim, a minimal dose of 60Gy is prescribed to any affected lnn.

### Treatment

The patient will be treated with the “PTV_EXIT” treatment plan, in 25 fractions. All treatments will be performed on an Elekta (Crawley, UK) Synergy linac with gantry mounted Cone Beam CT option. Daily Couch setup correction will be done after pre-treatment CBCT imaging consisting of M20 Field of View (i.e. 27,7 cm in SI direction), with bowtie filter, 120KV, 40 mA and 40 ms per frame, and 0,5 rpm gantry rotation speed. If necessary, adaptive treatment planning or plan-of-the-day is allowed.

Cisplatin 40 mg/m^2^ will be administered weekly during radiotherapy. In case of inadequate kidney function or other contra-indications, 5-FU or no chemotherapy is also allowed. Within 6 to 8 weeks after ending chemoradiation, a radical hysterectomy (Cibula classification: C1) will be performed [5].

### Imaging during and after treatment

All patients will undergo MRI at fixed time points (see Fig. 1 and Table 1). The MRI before, during and 2–3 weeks after CRT are considered routine standard procedures at our hospital. The MRI at the end of the treatment and within 1 week before HRT is supplemental for the study. The first is considered essential for adequate regression pattern analysis. The last MRI allows constructing a mold (e.g. 3D printing, widely available), which will act as a 3-dimentional rendering of the pre-HRT “uterine bed” and its slope. This allows mimicking the same position and angle rotation of the uterus as within the patient, which is necessary to allow for an adequate regression pattern analysis. After HRT, the HRT specimen itself will be scanned in the same position as within the patient, using the 3D-mold. The use of the 3D-mold will allow MRI-pathology comparison.

**Table 1:** MRI scan sequences

Timing MRI	Scan Sequence & used MRI device
Standard MRI before, during and 2–3 weeks after CRT	5 mm sagittal T2 Haste, 5 mm sagittal T2 TSE, 4 mm axial T2 TSE, 5 mm axial T1 GE, 4 mm axial DWI (b-values 0, 250, 500 and 1000 with calculated b 1400)
	at 1.5 Tesla MRI scanner (Siemens Aera, Erlangen, Germany)
Supplemental MRI at the end of treatment and pre-HRT	5 mm sagittal T2 Haste, 5 mm sagittal T2 TSE, 4 mm axial T2 TSE, 5 mm axial T1 GE, 4 mm axial DWI (b-values 50, 400 and 800 with calculated b 1400)
	at 3.0 Tesla MRI scanner (Siemens Trio or Prisma Fit, Erlangen, Germany)
Hysterectomyspecimen	1 mm coronal T2 Space with 1 mm sagittal and axial reconstructions, 4 mm axial DW (b-values 50, 250,500,750,1000 with calculated b 1400)
	at 3.0 Tesla MRI scanner (Siemens Trio or Prisma Fit, Erlangen, Germany)

*MRI* magnetic resonance imaging, *mm* millimeter, *TSE* Turbo Spin Echo, *GE* Gradient Echo, *DWI* diffusion weighted imaging

**Fig. 3:**
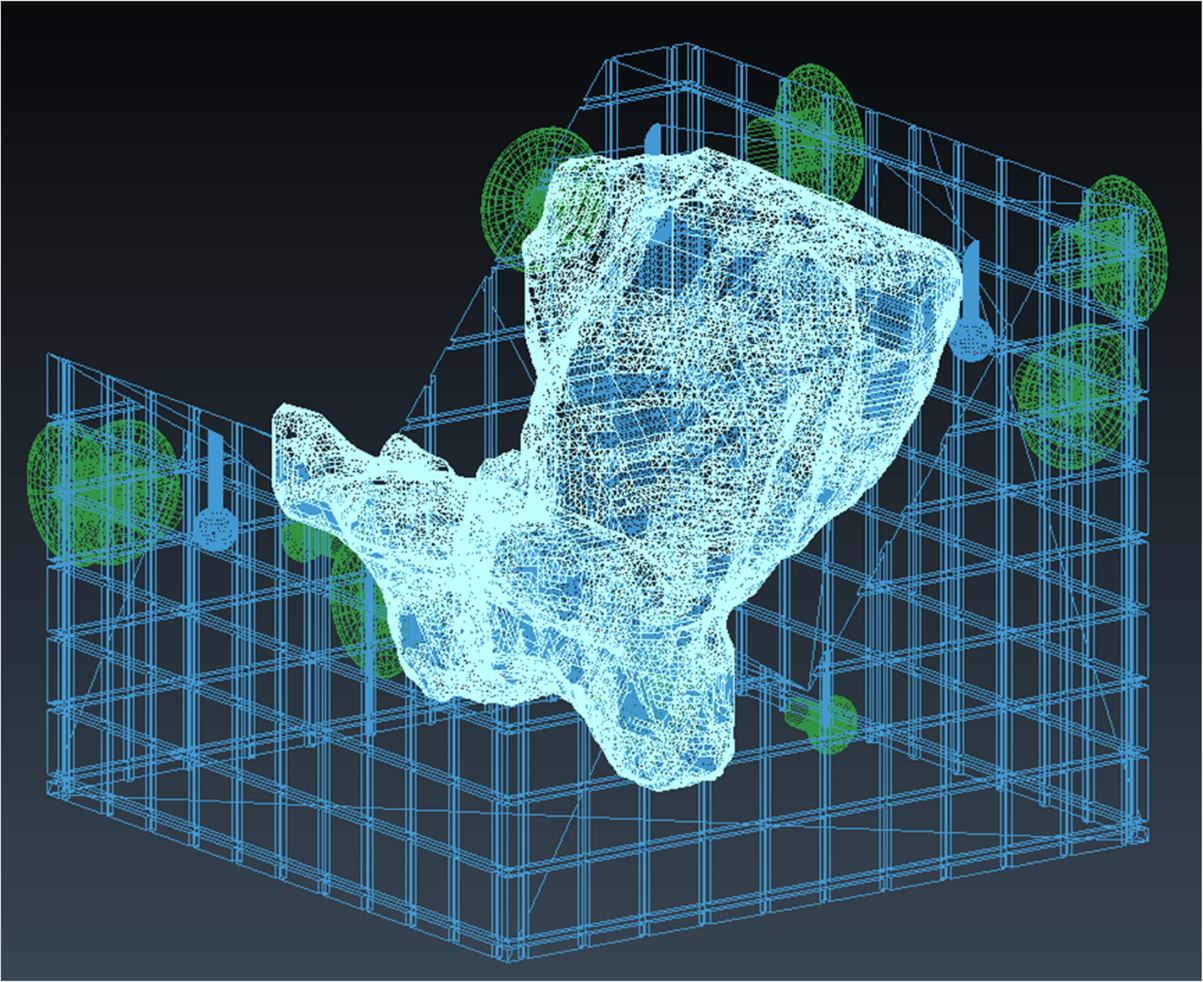
3D-mold. Example of a 3D-mold model created in Blender. The uterus, mold and knobs are depicted respectively in white, blue and green. The mold supports the uterus specimen, while the tissue is fixated to the knobs during sugery

The process to create the 3D mold starts with the conversion of a set of 2D contour slices delineated on the last MRI to a 3D volume using an in-house implemented marching cubes algorithm. The 3D volume is imported into Blender, an open-source 3D graphics software package. Around the imported volume, a box is generated using Python scripts and the uterus volume is subtracted from this box. The box is cutout further to support the lower half of the uterus specimen. On the side of the box, knobs are added to enable fixation of the tissue to the mold (Fig. 3).

### Clinicopathological examination

Upon receival, the fresh specimen will be removed from the 3D-mold and will be oriented, measured and weighed. The parametrial tissues, the soft tissue margins of the cervical canal and the vaginal cuff margin will be inked blue and green, signifying the left and right side, respectively. Parametria will be removed and entirely submitted for histological examination. Next, a probe will be inserted in the endocervical canal and the uterine cavity. The uterus and cervix will be bisected into posterior and anterior halves, to allow sufficient tissue fixation. These halves will be pinned on plastic to avoid curling of the tissue. The specimen will be inspected and described. Any macroscopically visible tumor will be measured and, if larger than 10 mm, a tissue sample of about 5 × 3 × 3 mm will be snap-frozen to allow future molecular studies. The bisected specimen will then be fixed in formalin for 24 h. After fixation, the vaginal cuff and the cervix will be transversally amputated and will be serially sectioned. These perpendicular clockwise sections will allow the assessment of the relationship of the tumor to the margin. Twelve, 3, 6 and 9 o’clock correspond with the anterior, left, posterior and right side of the cervix, respectively. Transverse sections of the endocervical canal and isthmus will be submitted for histological examination as well. The uterine corpus, fallopian tubes and ovaries will be macroscopically examined for abnormalities, and representative tissue samples will be submitted for histological examination. If initial hematoxylin and eosin (HE) stained tissue sections do not reveal any tumor rest in the cervix, multiple deeper sections will be performed. Additional immunohistochemistry for p40 and Cytokeratin5 will be performed to illustrate any residual isolated tumor cells when HE slides are deemed unclear. Any residual tumor, its localization and its relation to the surgical resection margins will be described in the report.

### Radiological-histological correlation

Transfer of the clinicopathological information to the post-HRT MRI will be done in cooperation with the pathologist and radiologist. In-house developed software in RayStation 6.0 allows correlation of tumor location in between the planning CT, the MRI scanned specimen and the HRT pathology result. Initially a gray based, region of interest constraint rigid fusion is done between the specimen and the planning CT, followed by a non-rigid hybrid deformable registration. Fusion of these MRIs with the planning-CT and treatment-plan will allow to allocate the remaining tumor in or out of the high-dose irradiated field.

### Follow-up

Acute toxicity will be scored conform previous studies (weekly during therapy, 10 days and 1 and 3 months after completing CRT) using a combination of RTOG supplemented with own grading for those toxicities not comprised in the RTOG scoring and CTCAE for hematologic toxicity [7, 17]. This will allow for matched-case comparison for acute toxicity with patients treated in previous studies [7, 17]. Chronic radiation toxicity (toxicity occurring > 3 months after completing CRT or acute toxicity lasting longer than 3 months) will be assessed at every follow-up visit conform previous studies [7, 17]. Again, this will allow for matched-case comparison for toxicity with patients treated in previous studies.

Local (LC), regional (RC) and distant control is defined as absence of disease at the primary tumor bed, the regional lymph nodes and distant sites respectively. Time to local relapse, regional relapse and distant relapse were defined as the time elapsed between biopsy and the first event (local, regional or distant relapse) or the last follow-up. PFS and OS were defined as the time elapsed between biopsy and any progression, death or the last follow-up.

### Additional and translational research

#### Planning study

Per patient, two different treatment plans (prescribed on PTV and PTV_EXIT as described above) will be generated. The OARs are the same for both plans and include small intestine, rectum, sigmoid colon, bladder and bone marrow. Prescription details can be found in our previous work [7, 8]. Dose Volume Histograms (DVHs) for all OARs will be calculated and compared between the two treatment plans. Per OAR, fixed dose-volume points of clinical relevance (e.g. volume of small bowel receiving ≥45 Gy, as used in previous research [8]) will be used for statistical comparison.

#### Correlation of high-risk regions on IMaging (DW-MRI) with pathology and regression pattern analysis (CRIMP)

The same patients who are the subjects of the clinical study will also be the subjects of the CRIMP study. The MRI at fixed time points (see Fig. 1) will be supplemented with diffusion weighing (DW). DW provides information about the amount of random movement of water molecules in a tissue. In healthy tissues the water molecules move relatively unhindered but in cancer the motion is strongly inhibited. This is depicted on DW as a high signal intensity area on high-b-value images with a corresponding low apparent diffusion coefficient (ADC) [18]. The ultimate aim of the CRIMP study is the correlation of tumoral ADC-values of the different DW-MRI with the pathology in order to predict therapy resistance or response to CRT at an early stage or even before start. The response on treatment assessed with pathology will be divided into 3 categories: A) no residual tumor or microscopic tumor rest consisting of fibrosis containing foci of neoplastic cells (the presence of isolated tumor cells/nests will not be considered as residual disease), B) macroscopic (> 7 mm) tumor rest revealing obvious tumor mass but showing CRT response and C) neoplastic tissue without any effect of radiation or regression. A high Apparent Diffusion Coefficient (ADC)_mean_ and limited increase (< 15%) in ADC_mean_ value 2 weeks after start of CRT corresponds with impaired response and survival [19, 20]. We hypothesize this will also correlate with residual tumor (isolated tumor cells excluded) at the HRT specimen. Both ADC_mean_ and median increase in ADC_mean_ will be correlated with the same pathology categories as mentioned here above.

### Statistical considerations

For the EXIT-trial; 21 patients are required to achieve a confidence interval with a half width of 11%, considering a negative predictive value of 98% for MRI [13] to predict absence of tumor (Wilson Score Confidence Interval for a Binomial Proportion). These patients will also be used for the observational CRIMP trial. For the plan comparison, statistics will be done using a paired T-test. All statistical analysis will be performed using SPSS.

### Ethical considerations

The study will be conducted in agreement with the Declaration of Helsinki. The protocol was approved by the Ethical Committee of Ghent University Hospital. All data collected for the purpose of research will be kept confidential. The patient’s identity will never be disclosed.

## Discussion

**Fig. 4:**
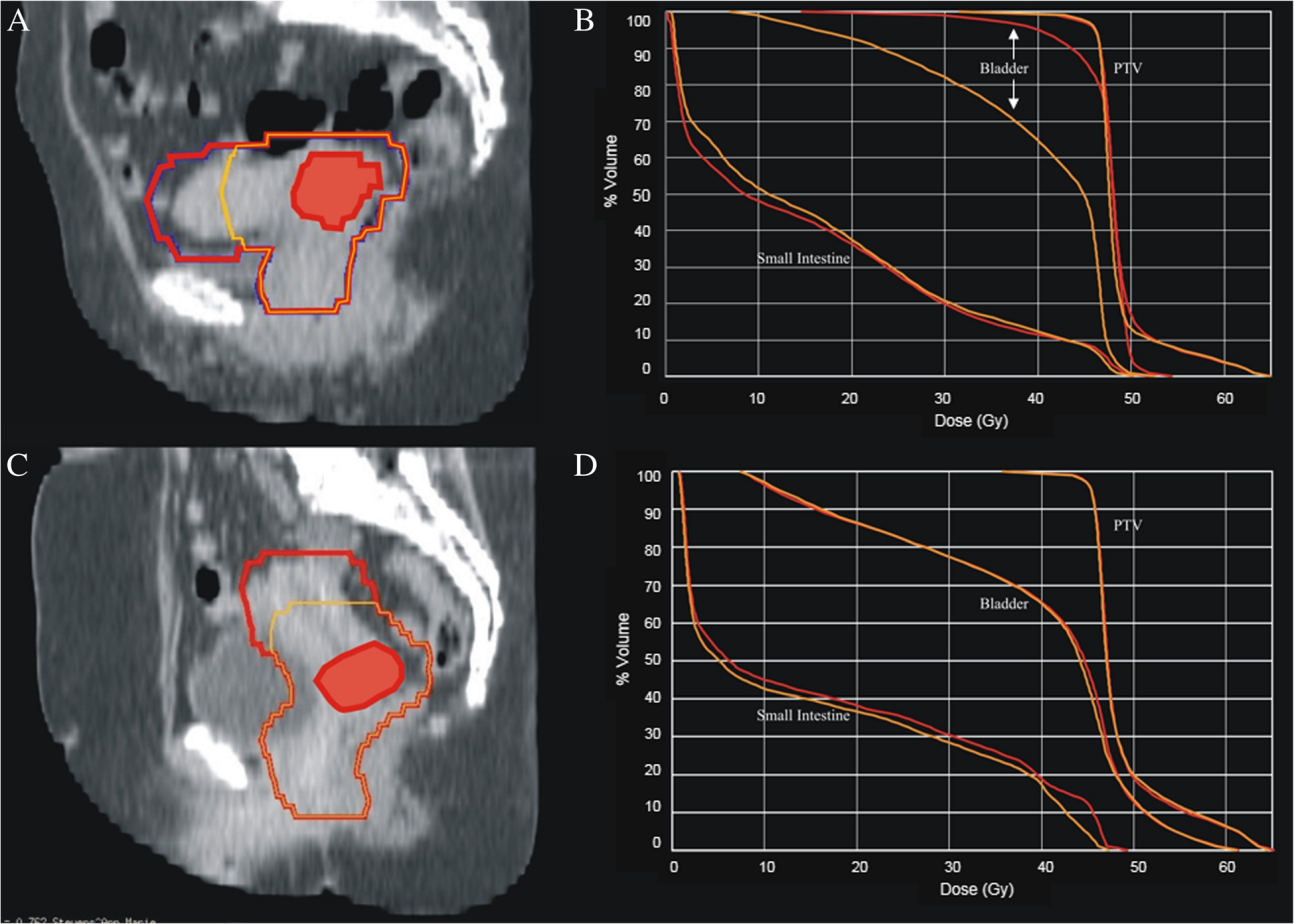
Dose Volume Histograms with and without the entire uterus included in the planning target volume. Dose Volume Histograms (DVHs) of 2 patients treated with a partial (yellow) or entire uterus (red) included in the planning target volume (PTV). **a** and **c** red contour = entire uterus included in the PTV; yellow contour = only the parts of the uterus closer than two cm of the gross tumor volume (GTV = red flooded contour) are included in the PTV. **c** and **d** DVHs of patient **a** and **c** respectively; the red DVHs correspond with the red contour (whole uterus); the yellow DVHs correspond with the yellow contour (selected parts of the uterus included in the CTV). The tail towards 62Gy in the DVH of the PTV corresponds with the SIB given to the GTV. In the first patient (**a**) the uterus lies in anteflexed position causing high doses to the bladder when irradiated entirely. A small reduction of PTV by reducing the amount of uterus included in the PTV causes a huge reduction in dose delivered to the bladder (**b**). A reduction of the PTV in case of a normal positioned uterus causes mainly a reduction in the irradiated volume of small bowel (**d**). Due to the large amounts of small bowel delineated, the reduction in terms of percentage seems small. However, in this case the amount of small bowel receiving 45Gy is reduced with 8%, which corresponds with 59 cc

Definitive chemoradiation is considered standard of care for LACC, wherein radiotherapy plays a crucial role. Unfortunately, toxicities caused by radiotherapy are frequent. Adding chemotherapy to conventional EBRT has even doubled the risk of severe acute hematological and gastro-intestinal toxicity and tripled platelet toxicity, in exchange for an improved OS [4]. Efforts to reduce this toxicity have only been successful in the last decades. As the understanding of radiation and radiation techniques improved, intensity modulated RT (IMRT) and arc techniques have become more prevalent and the standard-of-care to deliver EBRT in cervical cancer based on reduced gastro-intestinal (GI), genito-urinary (GU) and hematological toxicity [7, 17, 21–24]. Preliminary results from the only randomized trial comparing IMRT and conventional RT confirm a significant reduction in GI and GU toxicity “(https://clinicaltrials.gov/ct2/results?pg=1&load=cart&id=NCT01672892)”. The publication of the GEC-ESTRO recommendations for image-guided adaptive brachytherapy (IGABT) one decade ago was a significant step forward for the brachytherapeutic treatment of LACC. In the RetroEMBRACE study, the improved OS of 10% compared to historical series was accompanied with a limited severe (G3 or more) morbidity (3–6% per organ) [3, 25]. This is in agreement with other mono-institutional series, as is the observation that mild morbidity remains frequent with major impact on quality of life [3]. Hence, treatment related morbidity remains a major challenge. As the evolution in treatment techniques is doomed to reach its limits, other toxicity reducing efforts should be explored. The exquisite soft-tissue contrast of MRI has meant that the technique is having an increasing role in initial local staging, treatment planning (EBRT and IGABT) and for treatment monitoring of patients with advanced cervical cancer undergoing CRT. Despite the fact that MRI has been the imaging modality of choice for RT delineation [26], its ability to predict absence of tumor in the uterus has never been used to reduce treatment fields. Above that, there is no unequivocal evidence supporting the dogma that the whole uterus should be included in the CTV [26]. Despite the aforementioned reasoning, it remains to be proven that omitting unaffected parts of the uterus is safe, leads to lower dose to OARs (Fig. 4) reflecting in reduced toxicity.

At this moment, MRI is widely used for initial local staging and target/OAR delineation and also increasingly used for monitoring treatment response after CRT. However, the accuracy of conventional MRI techniques only (T2 sequences) is less than DW-imaging for monitoring treatment response [27, 28]. A clinical and iconographical complete response after CRT predicts a better outcome, whereas macroscopic residual disease at the HRT specimen is an adverse prognostic factor for survival [29]. Residual disease reflects radio-resistant tumor clones, which are more aggressive and eventually lead to cancer-related mortality. Overcoming this radioresistance with a more aggressive treatment and/or higher doses might improve outcome. However, not all tumors as well as not all intra-tumoral sub-regions require a more aggressive approach. Biological heterogeneity within a tumor is a well-recognized phenomenon, which critically influences treatment outcome [30]. Local failure is likely more adversely influenced by those at-risk sub-regions (tumor voxels) within the heterogeneous tumor, which possess unfavorable biological properties. Early assessment of tumor response and at-risk sub-regions would permit modification of strategy during therapy by escalating the dose on non-responsive regions within the tumor or de-escalating dose in responsive regions. In cervical cancer, several studies have shown the utility of DW-MRI in the prediction or monitoring of response to CRT or in the prediction of recurrent disease. DW-MRI can yield quantitative information by means of serial ADC-measurements [31]. Treatment-induced cell damage precedes morphological changes and is associated with increased ADC-values, which are reliable in predicting treatment response [27, 32]. Already 2 weeks after start of CRT, ADC_mean_ and the change in ADC_mean_ from the baseline correlate with the final MRI response [19, 33, 34] and with survival [20]. Liu et al. [33] found that also baseline ADC values are significantly lower for the patients achieving a complete radiological response than those achieving a partial response. Tumors with high baseline ADC are likely to be more necrotic than those with low baseline ADC, which in turn predicts poor outcomes related to hypoxia-mediated radio-resistance [35]. However, few tumors do not respond favorably to CRT despite having lower pretreatment ADC values [36]. This may be due to the fact that necrosis within a tumor is not always associated with a high ADC. In theory, coagulative necrosis without tumor cell liquefaction may not increase the ADC [36]. It is therefore essential to combine pretreatment ADC value with the change in this value after 2 weeks of treatment for response prediction. To date, studies have focused on ADC_mean_, based on a region of interest (ROI) defined as the whole tumor or on 1 slice of the whole tumor [37]. According to the above-mentioned findings, radioresistant regions within the tumor will have higher baseline ADC values [33] and less change in ADC at 2 weeks [19, 33, 34, 36]. We hypothesize that baseline ADC and its evolution 2 weeks [36] after start of CRT will correlate with tumor rest in the hysterectomy specimen and that future dose escalation to those “at-risk” regions within the GTV could reduce the amount of residual macroscopic disease. In this way, DW-MRI could be used for assessing early treatment response and functional tumor heterogeneity for a personalized cancer RT strategy.

## Abbreviations

3D: Three-Dimensional
5-FU: 5 Fluoro-Uracil
ADC: Apparent Diffusion Coefficient
AP: Antero-Posterior
C: Chemotherapy
CBCT: Cone Beam CT
CRT: Chemoradiotherapy
CTCAE: Common Terminology Criteria for Adverse Events
CTV: Clinical Target Volume
DVH: Dose Volume Histogram
DW: Diffusion Weighted
EBRT: External Beam Radiotherapy
GE: Gradient Echo
GI: Gastro-Intestinal
GTV: Gross Tumor Volume
GTV-prim: Gross Target Volume of the primary tumor
GU: Genito-Urinary
HE: Hematoxylin and Eosin
HRT: Hysterectomy
IGABT: Image Guided Adaptive Brachytherapy
IMAT: Intensity Modulated Arc Therapy
IMAT-C: Intensity Modulated Arc Therapy with Cisplatin
IMRT: Intensity Modulated Radiotherapy
KV: KiloVoltage
LACC: Locally Advanced Cervical Cancer
LC: Local Control
lnn: Lymph nodes
LR: Left-Right
mA: milliAmpere
MRI: Magnetic Resonance Imaging
ms: milliseconds
OAR(s): Organ(s) at Risk
OS: Overall Survival
18 FDG PET-CT: 18-fluorodeoxyglucose PET scan
PFS: Progression Free Survival
Prim: Primary tumor
PTV: Planning Target Volume
RC: Regional Control
RT: Radiotherapy
RTOG: Radiation Therapy Oncology Group
SI: Supero-inferior
SIB: Simultaneously Integrated Boost
TSE: Turbo Spin Echo
TV: Target Volume
